# Assessment of Physical Activity and Healthy Eating Behaviors Among US Adults Receiving Bariatric Surgery

**DOI:** 10.1001/jamanetworkopen.2022.17380

**Published:** 2022-06-16

**Authors:** Young-Rock Hong, Sandhya Yadav, Ryan Suk, Alexandra M. Lee, Faith A. Newsome, Crystal N. Johnson-Mann, Michelle I. Cardel, Kathryn M. Ross

**Affiliations:** 1 Department of Health Services Research, Management and Policy, College of Public Health and Health Professions University of Florida, Gainesville; 2 University of Florida Health Cancer Center, Gainesville; 3 Department of Management, Policy and Community Health, University of Texas Health Science Center at Houston School of Public Health, Houston; 4 Department of Kinesiology, Pennsylvania State University, University Park; 5 Department of Health Outcomes and Biomedical Informatics, University of Florida College of Medicine, Gainesville; 6 Department of Surgery, University of Florida College of Medicine, Gainesville; 7 WW International, Inc, New York, New York; 8 Department of Clinical and Health Psychology, College of Public Health and Health Professions University of Florida, Gainesville

## Abstract

**Importance:**

Bariatric surgery effectively treats severe obesity and metabolic diseases. However, individual outcomes vary depending on sustainable lifestyle change. Little is known about lifestyle patterns after bariatric surgery among the US population.

**Objective:**

To compare the level of physical activity and eating behavior among postbariatric surgery patients, individuals eligible for surgery, and those with normal weight.

**Design, Setting, and Participants:**

A cross-sectional study using nationally representative survey data from National Health and Nutrition Examination Survey 2015-2018. Respondents included for analysis were age 18 years or older, and categorized by individuals with normal weight, individuals who received bariatric surgery, and individuals clinically eligible for bariatric surgery. Analyses were performed from February to October 2021.

**Main Outcomes and Measures:**

Self-reported measures were used to assess physical activity (moderate-to-vigorous physical activity [MVPA], sedentary activity, and whether PA guidelines were met) and eating behaviors (total energy intake and Healthy Eating Index [HEI]–2015 diet quality scores).

**Results:**

Of 4659 study participants (mean [SD] age, 46.1 [18.6] years; 2638 [weighted percentage, 58.8%] women; 1114 [weighted percentage, 12.7%] Black, 1570 [weighted percentage, 68.6%] White), 132 (3.7%) reported that they had undergone any bariatric surgery. Median (IQR) time since surgery was 7 (3-10) years. After propensity-score weighting, individuals who underwent bariatric surgery reported more time spent in MVPA than those eligible for surgery (147.9 min/wk vs 97.4 min/wk). Among respondents with normal weight, 45.6% (95% CI, 40.8% to 52.4%) reported meeting PA guidelines, almost 2 times higher than those in the bariatric surgery (23.1%; 95% CI, 13.8% to 32.4%) or in the surgery-eligible group (20.3%; 95% CI, 15.6% to 25.1%). Propensity-score weighted overall HEI was higher for individuals with normal weight (54.4; 95% CI, 53.0 to 55.9) than those who underwent bariatric surgery (50.0; 95% CI, 47.2 to 52.9) or were eligible for the surgery (48.0; 95% CI, 46.0 to 50.0). Across all HEI components, mean scores were similar between the bariatric surgery and surgery-eligible groups. Total energy intake was the lowest among those who underwent bariatric surgery (1746 kcal/d; 95% CI, 1554 to 1937 kcal/d), followed by those with normal weight (1943 kcal/d; 95% CI, 1873 to 2013 kcal/d) and those eligible for bariatric surgery (2040 kcal/d; 1953 to 2128 kcal/d).

**Conclusions and Relevance:**

In this cross-sectional study, individuals who underwent bariatric surgery had beneficial lifestyle patterns compared with those eligible for surgery; however, these improvements seemed suboptimal based on the current guidelines. Efforts are needed to incorporate benefits of physical activity and a healthy, balanced diet in postbariatric care.

## Key Points

**Question:**

What are the differences in lifestyle patterns among individuals who received bariatric surgery compared with those eligible for surgery who did not receive it and those with normal weight?

**Findings:**

In this cross-sectional study of 4659 participants, postbariatric surgery patients reported more time spent on physical activity (50.6 min/wk) and lower total energy intake (−295 kcal/d) than those eligible for surgery, with levels of physical activity comparable with those with normal weight.

**Meaning:**

These results suggest that postoperative support for sustained behavioral changes is needed for postbariatric patients to help achieve long-term health benefits.

## Teaser

This cross-sectional study compares levels of physical activity and total energy intake of patients receiving bariatric surgery with eligible patients who have not had the operation and people with normal weight.

## Introduction

Obesity is a well-established risk factor for multiple chronic conditions, including metabolic syndrome, cardiovascular disease (CVD), and some cancers^1,2,3,4^; thus, the growing prevalence of obesity poses a serious public health concern. As of 2017-2018, obesity affects more than 42% of the US population, with 9% of adults having severe obesity (defined as BMI ≥40; calculated as weight in kilograms divided by height in meters squared).^3,4^ It has been estimated that nearly $210 billion can be attributable to obesity alone, and when considering obesity-associated chronic diseases, obesity accounts for an estimated $530 billion of US health care spending.^5^

Bariatric surgery is the most effective treatment for severe and complex obesity and metabolic diseases such as type 2 diabetes.^6,7,8^ However, a growing body of the literature suggests that long-term maintenance of health benefits requires long-term adherence to behavioral changes in physical activity and diet following surgery.^9,10,11^ According to the American Society for Metabolic and Bariatric Surgery (ASMBS), more than 200 000 individuals undergo bariatric surgery in the US each year, and this number is expected to grow.^12^ However, to date there is little known regarding physical activity and dietary patterns among individuals receiving bariatric surgery at the population level. Many patients receiving bariatric surgery maintain weight loss; however, about half of patients experience weight regain within 2 years and regain a range of 8 to 10 kg within 60 months compared with their lowest weight after the surgery overall.^13,14^ While regaining lost weight is not necessarily harmful, a deeper understanding of postoperative lifestyle patterns is needed to help inform preoperative education programs and postsurgical interventions to maximize the clinical benefits of bariatric surgery.^15,16^

Using a nationally representative sample of the US population, we examined and compared physical activity level, healthy eating behaviors, and total energy expenditure among US adults who underwent bariatric surgery relative to those who were eligible for, but did not receive, bariatric surgery and those with normal weight. We included the normal-weight group in addition to the nonbariatric surgery group in the study population to see if the bariatric surgery group had similar outcomes with the normal weight group (as a test for equivalency or normalization)^17,18^ and significantly different outcomes with the nonsurgery group.

## Methods

### Data Source

We conducted a cross-sectional study using data from the National Health and Nutrition Examination Survey (NHANES) for the years 2015 through 2018. The NHANES is a nationally representative survey that is conducted in 2-year cycles by the National Center for Health Statistics to assess the health and nutrition status of the US population.^19^ We combined 2 NHANES survey cycles (2015-2016 and 2017-2018) given the availability of information on bariatric surgery. More details about the sampling frame and data structure of NHANES have been documented elsewhere.^19^ In brief, NHANES data were gathered using household surveys, medical examinations, and laboratory tests. NHANES oversampled individuals aged 60 years or older, African American respondents, and Hispanic respondents and uses a multilevel weighting system to account for complex survey design and nonresponse. We adjusted for survey weights and followed recommended analysis strategy to generate nationally representative population estimates for the US.^20^ This study was deemed exempt from review by the University of Florida institutional review board because we used deidentified and publicly available data. To report our findings, we followed the Strengthening the Reporting of Observational Studies in Epidemiology (STROBE) reporting guideline.

### Study Population

Our initial sample included 11 848 participants aged 18 years or older. We categorized the individuals into 3 mutually exclusive groups: (1) individuals with normal weight (BMI range, 18.5-24.9), (2) individuals who received bariatric surgery (self-reported to have received bariatric surgery), and (3) individuals clinically eligible for bariatric surgery but reporting no receipt of bariatric surgery. Eligibility for bariatric surgery was identified according to the ASMBS recommendation of having a BMI of 40 or higher (severe obesity) or a BMI of 35 or higher with at least 1 obesity-related comorbidity (including hypertension, diabetes, chronic obstructive pulmonary disease [COPD], liver disease, hyperlipidemia, and any CVD).^21^ Those who did not fall in these groups (those with underweight BMI below 18.5, 188 respondents; did not meet the surgery eligibility, 6339 respondents) or with missing data on key study variables (662 respondents) were excluded (Table 1). The final study sample included 4659 participants.

**Table 1.** Characteristics of Study Participants

Characteristics	Respondents, No. (weighted %)^a^			*P* value	Propensity-score weighted respondents, No. (weighted %)^b^			*P* value	*P* value bariatric vs eligible group
	Normal weight (n = 2906)	Had bariatric surgery (n = 132)	Eligible but no surgery (n = 1621)		Normal weight (n = 2403)	Had bariatric surgery (n = 121)	Eligible but no surgery (n = 1496)		
BMI, mean (SD)	22.4 (2)	35.6 (7)	41.9 (6)	<.001	NA	NA	NA	NA	NA
Mean (SD) age, y	43.8 (20)	52.2 (12)	49.6 (16)	<.001	51.8 (19)	52.8 (12)	53.9 (16)	.18	.45
Sex									
Women	1536 (57.2)	105 (78.5)	997 (59.8)	<.001	1266 (78.4)	96 (77.5)	915 (76.5)	.95	.85
Men	1370 (42.8)	27 (21.5)	624 (40.2)		1137 (21.6)	25 (22.5)	581 (23.5)		
Race									
Non-Hispanic Black	576 (10.3)	40 (12.7)	498 (16.2)	<.001	472 (8.9)	36 (12.7)	456 (18.1)	.17	.32
Non-Hispanic White	969 (64.7)	51 (69.3)	550 (62.2)		860 (71.5)	48 (69.6)	525 (66.3)		
Other^c^	1361 (25.0)	41 (18.0)	573 (21.5)		1071 (19.6)	37 (17.7)	515 (15.6)		
Education									
High school or less	1106 (32.8)	40 (25.2)	704 (37.9)	<.001	990 (21.9)	37 (26.2)	664 (31.1)	.09	.30
Some college	721 (28.2)	53 (28.2)	601 (38.0)		661 (23.9)	48 (29.1)	573 (34.9)		
College ≥4 y	831 (39.0)	39 (46.6)	282 (24.1)		752 (54.2)	36 (44.7)	259 (34.0)		
Family income^d^									
Low	866 (22.9)	29 (15.1)	529 (23.8)	.08	683 (14.3)	27 (16.7)	472 (19.3)	.85	.85
Middle	680 (18.3)	26 (11.7)	422 (20.0)		568 (10.8)	23 (11.5)	391 (12.4)		
High	1340 (58.8)	77 (73.2)	670 (56.2)		1152 (74.9)	71 (71.8)	633 (68.2)		
Health insurance									
Private	1196 (51.0)	65 (55.6)	557 (44.6)	.006	977 (53.6)	57 (51.4)	532 (48.9)	.98	.95
Public	1247 (35.0)	58 (35.7)	829 (43.2)		1050 (37.1)	56 (39.2)	751 (41.5)		
Uninsured	457 (14.0)	9 (8.7)	232 (12.2)		376 (9.3)	8 (9.4)	213 (9.6)		
Self-reported fair/poor general health	406 (10.8)	33 (22.2)	665 (36.2)	<.001	384 (11.2)	33 (23.3)	648 (33.7)	.001	.11
Mobility limitation	251 (6.4)	36 (26.2)	486 (23.9)	<.001	225 (9.7)	33 (26.4)	455 (28.1)	.007	.84
Current smoking	586 (18.1)	18 (24.3)	273 (29.7)	<.001	523 (18.8)	18 (16.8)	254 (14.4)	.77	.49
CVD	224 (5.5)	19 (11.8)	243 (12.1)	<.001	204 (11.3)	17 (12.7)	224 (16.5)	.46	.37
Cancer	240 (9.5)	15 (15.5)	156 (11.6)	.21	227 (23.7)	14 (14.0)	152 (17.0)	.11	.58
Diabetes	203 (4.0)	37 (23.9)	471 (24.0)	<.001	185 (7.6)	37 (25.7)	448 (33.7)	<.001	.10
Hypertension	633 (17.2)	83 (56.3)	993 (58.3)	<.001	560 (31.7)	77 (59.5)	927 (70.1)	<.001	.14
Hyperlipidemia	693 (22.7)	56 (48.9)	744 (45.2)	<.001	628 (46.0)	52 (51.2)	701 (60.8)	.16	.19
COPD	103 (3.1)	5 (7.1)	107 (5.7)	.007	97 (6.8)	5 (7.8)	100 (7.4)	.96	.92

Abbreviations: BMI, body mass index (calculated as weight in kilograms divided by height in meters squared); COPD, chronic obstructive pulmonary disease; CVD, cardiovascular disease; NA, not applicable.

^a^ Percentages weighted to be nationally representative and account for nonresponse.

^b^ Propensity-score weighted estimates, adjusted for all characteristics listed in the Table except BMI.

^c^ Includes Asian, Hispanic, and multiple races.

^d^ Based on Federal Poverty Level: low income, Federal Poverty Level <200%; middle, Federal Poverty Level 200%-399%; high, Federal Poverty Level >399%.

### Outcome Variables

Our primary outcomes were physical activity levels, diet quality, and total energy intake. Self-reported physical activity measure was divided into 3 levels—moderate-to-vigorous physical activity (MVPA), sedentary activity, and meeting physical activity guidelines.^22^ We calculated the minutes per week of vigorous physical activity by multiplying the number of days of vigorous physical activity in a week by minutes of vigorous physical activity in a day. Likewise, we calculated the minutes per week of moderate physical activity by multiplying the number of days of moderate physical activity in a week by minutes of moderate physical activity in a day. Total MVPA was calculated by summing the minutes per week of vigorous physical activity and moderate physical activity. Self-reported sedentary activity was recorded in minutes per day. Compliance with physical activity guidelines was defined as engaging in at least 150 minutes a week of moderate-intensity or 75 minutes a week of vigorous-intensity aerobic physical activity.^22^

To analyze diet quality, we merged 24-hour dietary recall data collected in NHANES with the USDA Food Patterns Equivalents database for years 2015-2016 and 2017-2018 and calculated Healthy Eating Index–2015 (HEI) scores.^23,24^ This measure included 13 dietary components; 9 were adequacy components (total vegetables, greens and beans, total fruits, whole fruits, whole grains, dairy, total protein foods, seafood and plant proteins, and saturated fatty acids) and 4 were moderation components (sodium, refined grain, saturated fat, and added sugar). Higher intake of adequacy components resulted in higher HEI scores, which corresponded to a healthier diet. Conversely, higher intake of moderation components resulted in lower scores, corresponding to a less healthy diet. An overall HEI score ranging from 0 to 100, with higher scores indicating better diet quality, was calculated by adding the 13 component scores. More details on the HEI scoring standards can be found in Reedy et al.^24^ We considered a 3-point or more difference in overall HEI score as a clinically meaningful difference, consistent with previous studies.^25,26^ Total energy intake (kilocalories per day) was calculated using NHANES dietary interview data.^19,27^ In brief, all variables labeled energy (kilocalories) in the total nutrient intakes files were summed to estimate total energy expenditure, including nutrients obtained from foods, beverages, and water (tap and bottled water).^27^

### Matching Variables

Considering disparities in access to bariatric surgery (eg, by race and ethnicity and other socioeconomic status),^28^ we used propensity score weighting to address selection bias and confounding between receipt of bariatric surgery and outcomes of interest while retaining all study participants in the analysis.^29,30^ Covariates used in adjustments included participants’ age, self-reported race and ethnicity (non-Hispanic Black, non-Hispanic White, or other [Asian, Hispanic, and multiple races]), education (high school or less, some college, college 4 years or more), family income (low, below 100% of federal poverty level [FPL]; middle, 100%-200% of FPL; high, above 200% of FPL), health insurance (private, public, uninsured), general health status (excellent or good, fair or poor), current smoking status, mobility limitation (difficulty walking), diagnosis of cardiovascular disease (CVD), cancer, diabetes, hypertension, hyperlipidemia, and COPD. We aggregated self-identified Hispanic, Asian, or multiracial participants into other race and ethnicity group because of small sample size. To create a propensity score for receiving bariatric surgery, we used a survey design–adjusted logistic regression procedure.

### Statistical Analysis

To compare baseline characteristics across surgery and BMI groups, we used first-order Rao-Scott χ^2^ tests. We fit multiple general linear models to examine the mean of physical activity levels (minutes per week of MVPA, minutes per day of sedentary activity, and proportion of meeting physical activity guidelines), HEI scores, and total energy intake between the 3 study groups. Each study outcome was examined in a separate model. SAS PROCSURVEY procedures were used for population estimation, which included weight, cluster, and strata statements and incorporated sampling weights. We present both unweighted and propensity-weighted analysis results for the study groups; percentages reflect weighted estimates (either for sample weight or propensity weight). Statistical significance was estimated at *P* < .05 in 2-sided tests, and all analyses were conducted using SAS software, version 9.4 (SAS Institute, Inc).

## Results

### Sample Characteristics

Of 4659 study participants (mean [SD] age, 46.1 [18.6] years old; 2638 [58.8%] women; 1114 [12.7%] Black, 1570 [68.6%] White), 132 (3.7%, representing 3.6 million US adults) reported that they had undergone any type of bariatric surgery. Median (IQR) time since the surgery was 7 (3-10) years. Compared with individuals eligible for bariatric surgery but who did not undergo surgery, individuals who underwent bariatric surgery tended to be older, female, or identified as White (Table 1). Compared with those with normal weight, those eligible for or who underwent bariatric surgery were more likely to report fair or poor general health (33 of 132 [22.2%] vs 406 of 2906 [10.8%]), current smoking (18 [24.3%] vs 586 [18.1%]), mobility limitations (36 [26.2%] vs 251 [6.4%]), or more chronic conditions (diabetes, 37 [23.9%] vs 203 [4.0%]; CVD, 19 [11.8%] vs 224 [5.5%]). After applying propensity score-weighting, the differences between the groups were largely mitigated except for general health status and some chronic conditions (diabetes and hypertension). All standardized differences between the surgery group and the surgery-eligible group were minimal.

### Physical and Sedentary Activity

After propensity-score weighting, individuals who underwent bariatric surgery reported more time spent in MVPA than those eligible for surgery (147.9 min/wk; 95% CI, 101.6 to 194.4 min/wk vs 97.4 min/wk; 95% CI, 82.6 to 112.2 min/wk) (Table 2). For sedentary activity, there was no significant difference between individuals in the bariatric surgery group and those in the surgery-eligible group. Of those with normal weight, 45.6% (95% CI, 40.8% to 52.4%) were estimated to meet PA guidelines, which was almost 2 times higher than those in the bariatric surgery (23.1%; 95% CI, 13.8% to 32.4%) or in the surgery-eligible group (20.3%; 95% CI, 15.6% to 25.1%).

**Table 2.** Comparison of PA Levels Between Normal Weight, Bariatric Surgery, and Surgery-Eligible Groups

Outcomes	Estimate (95% CI)			Global *P* value	Propensity-score weighted estimate (95% CI)			Global *P* value
	Normal weight	Had bariatric surgery	Eligible but no surgery		Normal weight	Had bariatric surgery	Eligible but no surgery	
MVPA, min/wk	225.1 (201.0 to 249.3)	130.6 (92.5 to 168.7)	113.2 (99.4 to 127.1)	<.001	191.1 (166.7 to 215.5)	147.9 (101.6 to 194.4)	97.4 (82.6 to 112.2)	<.001
Difference^a^	[Reference]	–94.6 (–134.9 to –54.2)^b^	–111.9 (–139.6 to –84.2)^b^	NA	[Reference]	–43.1 (–94.4 to 8.2)	–93.7 (–122.2 to –65.2)^b^	NA
*P* value^a^	NA	<.001	<.001	NA	NA	.14	<.001	NA
Difference^a^	111.9 (84.2 to 139.6)^b^	17.3 (–23.9 to 58.5)	[Reference]	NA	93.7 (65.2 to 122.1)^b^	50.6 (1.4 to 99.8)^b^	[Reference]	NA
*P* value^a^	<.001	.40	NA	NA	<.001	.04	NA	NA
Sedentary activity, min/d	349.7 (335.9 to 363.4)	412.9 (351.9 to 474.0)	423.8 (406.6 to 441.0)	<.001	350.7 (332.7 to 368.9)	404.3 (332.1 to 476.4)	433.2 (412.6 to 453.8)	<.001
Difference^a^	[Reference]	63.3 (4.5 to 122.0)^b^	74.1 (54.3 to 93.9)^b^	NA	[Reference]	53.6 (–18.1 to 125.4)	82.5 (56.2 to 108.9)^b^	NA
*P* value^a^	NA	.03	<.001	NA	NA	.22	<.001	NA
Difference^a^	–74.1 (–93.9 to –54.3)^b^	–10.8 (–63.1 to 41.5)	[Reference]	NA	–82.5 (–108.9 to –56.2)^b^	–28.9 (–100.7 to 42.9)	[Reference]	NA
*P* value^a^	<.001	.68	NA	NA	<.001	.20	NA	NA
Meeting PA guidelines, %^c^^,^^d^	44.8 (40.3 to 49.3)	25.9 (16.1 to 35.8)	22.9 (18.8 to 26.9)	<.001	45.6 (40.8 to 52.4)	23.1 (13.8 to 32.4)	20.3 (15.6 to 25.1)	<.001
Difference^a^	[Reference]	–18.8 (–26.9 to –10.9)^b^	–21.9 (–28.3 to –15.6)^b^	NA	[Reference]	–23.5 (–33.3 to –13.7)^a^	–26.2 (–34.4 to –18.0)^b^	NA
*P* value^a^	NA	<.001	<.001	NA	NA	<.001	<.001	NA
Difference^a^	21.9 (15.6 to 28.3)^b^	3.1 (–14.6 to 8.4)	[Reference]	NA	26.2 (18.0 to 34.4)^b^	2.8 (–13.9 to 8.5)	[Reference]	NA
*P* value^a^	<.001	.71	NA	NA	<.001	.73	NA	NA

Abbreviations: MVPA, moderate-to-vigorous physical activity; NA, not applicable; PA, physical activity.

^a^ Post hoc between-group comparisons.

^b^ Significant difference from the reference group.

^c^ Percentages weighted to be nationally representative and account for nonresponse.

^d^ Defined as meeting guidelines if engaged in at least 150 minutes a week of moderate-intensity or 75 minutes a week of vigorous-intensity aerobic physical activity.

### Healthy Eating and Total Energy Intake

Propensity-score weighted overall HEI score was higher for individuals with normal weight (54.4; 95% CI, 53.0 to 55.9) than those who underwent bariatric surgery (50.0; 95% CI, 47.2 to 52.9; *P* = .01) or were eligible for the surgery (48.0; 95% CI, 46.0 to 50.0; *P* < .001) (Table 3). Across all HEI components, mean scores were similar between the bariatric surgery and surgery-eligible groups. Total energy intake was the lowest among those who underwent bariatric surgery (1746 kcal/d; 95% CI, 1554 to 1937 kcal/d), followed by those with normal weight (1943 kcal/d; 95% CI, 1873 to 2013 kcal/d) and those eligible for bariatric surgery (2040 kcal/d; 95% CI, 1953 to 2128 kcal/d).

**Table 3.** Comparison of HEI Component and Total Energy Expenditure Between Normal Weight, Bariatric Surgery, and Surgery Eligible Groups

Outcomes	HEI, Max Score	Estimate (95% CI)			Global *P* value	Propensity-score weighted estimate (95% CI)			Global *P* value
		Normal weight	Had bariatric surgery	Eligible but no surgery		Normal weight	Had bariatric surgery	Eligible but no surgery	
Total vegetables	5	3.1 (2.9 to 3.2)	2.7 (2.4 to 3.0)	2.9 (2.8 to 3.1)	.03	3.4 (3.2 to 3.5)	2.8 (2.5 to 3.1)	3.1 (2.8 to 3.3)	<.001
Greens and beans	5	1.8 (1.6 to 1.9)	1.6 (0.6 to 2.4)	1.5 (1.3 to 1.7)	.03	2.0 (1.7 to 2.2)	1.4 (0.5 to 2.4)	1.6 (1.3 to 1.8)	.04
Total fruits	5	2.2 (2.0 to 2.4)	1.8 (1.1 to 2.5)	1.7 (1.5 to 1.8)	<.001	2.3 (2.1 to 2.6)	1.8 (1.1 to 2.4)	1.8 (1.5 to 2.1)	.008
Whole fruits	5	2.3 (2.1 to 2.5)	1.6 (1.0 to 2.2)	1.8 (1.6 to 2.0)	<.001	2.5 (2.2 to 2.8)	1.6 (1.0 to 2.1)	2.0 (1.6 to 2.3)	.005
Whole grains	10	2.7 (2.4 to 3.0)	2.9 (1.7 to 4.1)	2.1 (1.9 to 2.4)	.02	3.0 (2.6 to 3.4)	3.0 (1.8 to 4.2)	2.3 (1.9 to 2.7)	.03
Dairy	10	4.8 (4.6 to 5.1)	4.1 (3.0 to 5.3)	4.5 (4.3 to 4.8)	.08	4.9 (4.5 to 5.2)	4.3 (3.3 to 5.4)	4.5 (4.1 to 4.9)	.21
Total protein foods	5	4.2 (4.1 to 4.3)	4.1 (3.7 to 4.5)	4.3 (4.2 to 4.3)	.34	4.2 (4.1 to 4.3)	4.2 (3.9 to 4.5)	4.2 (4.1 to 4.4)	.70
Seafood and plant proteins	5	2.6 (2.5 to 2.8)	2.2 (1.4 to 3.1)	2.2 (1.9 to 2.4)	.003	2.8 (2.6 to 3.0)	2.1 (1.3 to 2.9)	2.3 (2.0 to 2.6)	.007
Fatty acids	10	5.1 (4.8 to 5.4)	6.0 (5.3 to 6.7)	4.7 (4.4 to 5.1)	.01	5.1 (4.7 to 5.5)	5.9 (5.2 to 6.5)	4.7 (4.2 to 5.2)	.03
Sodium	10	4.5 (4.3 to 4.7)	3.9 (3.0 to 4.8)	3.9 (3.6 to 4.2)	.002	4.6 (4.3 to 4.9)	4.0 (3.1 to 4.8)	4.0 (3.6 to 4.4)	.07
Refined grain	10	6.5 (6.2 to 6.7)	6.6 (5.3 to 7.9)	5.8 (5.5 to 6.2)	.01	7.0 (6.7 to 7.3)	6.3 (5.1 to 7.6)	5.8 (5.3 to 6.4)	.002
Saturated fat	10	5.8 (5.6 to 6.0)	6.0 (5.2 to 6.8)	5.0 (4.7 to 5.3)	<.001	5.6 (5.3 to 5.9)	5.8 (5.0 to 6.6)	4.9 (4.5 to 5.3)	.01
Added sugar	10	7.0 (6.7 to 7.2)	7.0 (6.0 to 8.0)	6.7 (6.4 to 7.0)	.25	7.1 (6.7 to 7.5)	6.8 (5.8 to 7.9)	6.8 (6.5 to 7.2)	.44
Overall HEI	100	52.5 (51.0 to 53.9)	50.4 (47.6 to 53.3)	47.1 (45.7 to 48.5)	<.001	54.4 (53.0 to 55.9)	50.0 (47.2 to 52.9)	48.0 (46.0 to 50.0)	<.001
Difference^a^	NA	[Reference]	–2.0 (–5.7 to 1.7)	–5.4 (–6.6 to –4.1)^b^	NA	[Reference]	–4.4 (–7.8 to –1.0)^b^	–6.4 (–8.5 to –4.4)^b^	NA
*P* value^c^	NA	NA	.28	<.001	NA	NA	.01	<.001	NA
Difference^a^	NA	5.4 (4.1 to 6.6)^b^	3.3 (0.5 to 6.6)^b^	[Reference]	NA	6.4 (4.4 to 8.5)^b^	2.0 (–1.3 to 5.4)	[Reference]	NA
*P* value^c^	NA	<.001	.047	NA	NA	<.001	.23	NA	NA
Total energy intake, kcal/d	NA	2122 (2054 to 2190)	1687 (1486 to 1888)	2145 (2083 to 2207)	<.001	1943 (1873 to 2013)	1746 (1554 to 1937)	2040 (1953 to 2128)	.002
Difference^a^	NA	[Reference]	–435 (–640 to –229)^b^	23 (–59 to 106)	NA	[Reference]	–197 (–392 to –2)^b^	98 (–3 to 199)	NA
*P* value^c^	NA	NA	<.001	.57	NA	NA	.047	.06	NA
Difference^a^	NA	–23 (–106 to 59)	–458 (–643 to –273)^b^	[Reference]	NA	–98 (–199 to 3)	–295 (–466 to –124)^b^	[Reference]	NA
*P* value^c^	NA	.57	<.001	NA	NA	.06	.001	NA	NA

Abbreviations: HEI, Healthy Eating Index; NA, not applicable.

^a^ Due to inadequate sample size issue, post hoc comparison was conducted only for overall HEI not each dietary component.

^b^ Significant difference from the reference group.

^c^ Post hoc between-group comparisons.

## Discussion

Using a nationally representative sample of US adults, we found that individuals who underwent bariatric surgery reported more time spent in physical activity (36 minutes more in a typical week) and had lower total energy intake (294 kcal/d lower) than those who were surgery-eligible but did not receive surgery. However, further analysis showed that receipt of bariatric surgery was not associated with meeting PA guidelines or higher dietary quality (as measured by HEI). Taken together, our findings suggest that long-term weight loss achieved through bariatric surgery is unlikely to result in individuals meeting the current minimum recommended levels of MVPA (over 150 min/wk) or diet quality recommendations and likely reflect significant physiological changes and reductions in energy intake. Further intervention from health care professionals may be needed to support higher levels of physical activity and diet quality in postoperative bariatric patients.

Change in physical activity and sedentary behavior after bariatric surgery is a growing area of interest.^31,32,33^ Although earlier studies focused on postsurgery physical activity in the short-term,^32,33,34,35^ a novelty and strength of our study was that it captured physical activity in varying time frames (median 7 years) after bariatric surgery in the general population. More in-depth understanding of these lifestyle behaviors seems to be needed to maximize the long-term health benefits of surgical weight reduction. Our findings appear to concur with previous studies that show improvements in active time following bariatric surgery, given that the surgery group had a higher mean MVPA than the surgery-eligible group.^31,36^ However, it is important to consider the activity intensity and postsurgery time frame, as these may affect the interpretation of our results. For example, a 2016 study by Afshar and colleagues^36^ found no significant change in physical activity before and 6 months after bariatric surgery using subjective and device-based measures of physical activity. Surgical recovery may impede engagement in MVPA while the body is healing and the patient is adjusting to new eating patterns. A longitudinal study^37^ conducted in bariatric surgery patients found that 47% of postoperative bariatric patients were sedentary or somewhat active, while 14% were active and 6% were highly active. In those studies, participants mainly reported participating in physical activity through activities in daily living (eg, playing with children, gardening) rather than structured physical activity regimens, suggesting the importance of assessing daily activities as a proxy for physical activities in patients who have undergone bariatric surgery. For example, measuring total physical activity volume throughout the day, like step counts, may be a more useful indicator of activity in this patient population given the focus of recent physical activity guidelines.^38^

There are a few factors that may explain lower physical activity rates in bariatric surgery patients compared with patients with normal weight. First and foremost, patients with obesity are more likely to be stigmatized while participating in physical activity.^39,40^ Second, a recent study suggested that physical activity recommendations applicable to the general population may not be realizable for postbariatric surgery patients because of persistent physical and mental health challenges that remain after bariatric surgery.^36,41^ Additional support for setting realistic goals and achieving behavioral change within the domain of physical activity (eg, incorporating lessons on SMART [Specific, Measurable, Achievable, Relevant, and Time-bound] goals) may be beneficial components for both preoperative and postoperative education programs.^42^ More substantive, direct, intentional work is necessary to promote dietary and lifestyle behaviors among postbariatric patients.

Although not statistically significant, we showed a potentially meaningful difference in sedentary behavior between those who underwent surgery and those who were eligible but did not, which may show a decrease in sedentary behavior (36 minutes less per day) and a corresponding increase in light intensity physical activity for those who underwent surgery. Vatier and colleagues^43^ also found a decrease in sedentary behavior after bariatric surgery independent of differences in physical activity, and suggested that this may be a result of favorable changes in body composition. The latest PA guidelines suggest “a strong relationship between time in sedentary behavior and the risk of all-cause mortality and cardiovascular disease mortality in adults.”^38^ Because the risk related to sedentary behavior is not dependent on the duration of MVPA, a decrease in prolonged periods of sedentary behavior would be an important goal for the postbariatric population. Future research studying activity behavior in persons who underwent bariatric surgery should measure sedentary behavior time, physical activity achieved through daily activities, bouts of prolonged sedentary time, and light-intensity physical activity, as these may be more relevant indicators of behavior change for this population. Future research should also investigate telemonitoring and use of mobile health lifestyle applications (known as *mHealth*) as interventions to increase physical activity and weight maintenance after bariatric surgery.

Our findings also indicated the lowest daily energy intake among bariatric surgery patients compared with respondents with normal weight and surgery-eligible respondents. However, overall HEI scores of patients undergoing bariatric surgery were lower than respondents with normal weight. Although studies have documented misreporting or underreporting of dietary consumption among individuals with obesity,^44,45^ changes in energy intake and diet quality after bariatric surgery may be because of alterations in the gut following bariatric surgery, which may result in physiological changes (such as “dumping syndrome” or feeling ill after consuming foods high in fat or sugar) that affect food consumption or adherence to certain dietary patterns.^31,46^ Additionally, the eating index is based on achieving recommended levels of portion sizes for specific food groups, which may be challenging to consume given the physiological changes to the gut that limit food consumption. This also results in overall energy intake being lower because all bariatric procedures have a restrictive component.^11,47,48,49^ In the long term, however, these changes could revert to presurgical levels and possibly cause an increase in energy intake over time.^50^ Taken together, our findings suggest that postsurgical patients may need more continual support for engaging in healthy lifestyle behaviors to maintain the effects of the surgery.

### Strengths and Limitations

The primary strength of this study was that we used data from a recent nationally representative sample from the NHANES (representing 100 million US adults), which included validated measures of diet and physical activity-related assessments. Nevertheless, this study has several limitations. First, we were not able to run analyses that incorporated time since the surgery date due to the manner in which this would limit the sample size and generate unreliable estimates.^51^ Given that most weight regain occurs between 2 and 5 years after surgery,^13,14^ additional studies with a greater sample size are needed to further understand lifestyle patterns during early postsurgery period and long-term health outcomes.^15,16^ Second, information on the type of surgery (eg, gastric sleeve, gastric bypass, duodenal switch) was not available in the NHANES data. Third, since we used self-reported data, our findings may be subject to recall bias. For example, self-reported MVPA data often have a right-skewed distribution, resulting in overreporting of MVPA. Thus, average minutes of physical activity reported should be interpreted with caution. Moreover, we relied on self-reported 24-hour dietary recall data to assess energy intake. Although NHANES dietary data are validated and considered reliable,^23,24^ it may be inadequate to assess total usual energy intake because of the timeframe of measure (eg, seasonal variation) and variations in individual intake.^52^ Fourth, this was a cross-sectional study, which limited causal inference of study findings. Lastly, we used the propensity score-weighting approach to minimize the selection bias of receipt of bariatric surgery^29,30^; however, this was based on available data, and we could not adjust for unobserved factors (eg, insurance coverage, provider information). Future studies with longitudinal data are warranted to confirm findings in this study.

## Conclusions

The current study examined physical activity and eating behavior in a representative sample of US adults who either were normal weight with no history of bariatric surgery, underwent bariatric surgery, or were eligible for bariatric surgery but had not undergone the procedure. Results demonstrated that individuals who underwent bariatric surgery showed higher engagement in physical activity and lower total energy intake compared with those eligible for the surgery. However, these improvements were not aligned with the current physical activity recommendations. By incorporating behavior change strategies that focus on reducing sedentary behavior and increasing total physical activity volume, bariatric programs could achieve better long-term benefits for this unique population.
